# Clobazam and Its Active Metabolite *N*-desmethylclobazam Display Significantly Greater Affinities for α_2_- versus α_1_-GABA_A_–Receptor Complexes

**DOI:** 10.1371/journal.pone.0088456

**Published:** 2014-02-12

**Authors:** Henrik Sindal Jensen, Kathryn Nichol, Deborah Lee, Bjarke Ebert

**Affiliations:** 1 Synaptic Transmission, Neuroscience Drug Discovery, H. Lundbeck A/S, Valby, Denmark; 2 Medical Affairs, Lundbeck LLC, Deerfield, Illinois, United States of America; 3 Clinical Affairs, Lundbeck LLC, Deerfield, Illinois, United States of America; 4 Department of Electrophysiology, H. Lundbeck A/S, Valby, Denmark; McLean Hospital/Harvard Medical School, United States of America

## Abstract

Clobazam (CLB), a 1,5-benzodiazepine (BZD), was FDA-approved in October 2011 for the adjunctive treatment of seizures associated with Lennox-Gastaut syndrome (LGS) in patients 2 years and older. BZDs exert various CNS effects through allosteric modulation of GABA_A_ receptors. The structurally distinct, 1,4-BZD clonazepam (CLN) is also approved to treat LGS. The precise mechanisms of action and clinical efficacy of both are unknown. Data show that the GABA_A_ α_1_-subunit–selective compound zolpidem [ZOL] exhibits hypnotic/sedative effects. Conversely, data from knock-in mice carrying BZD binding site mutations suggest that the α_2_ subunit mediates anticonvulsant effects, without sedative actions. Hence, the specific pattern of interactions across the GABA_A_ receptor complexes of BZDs might be reflected in their clinical efficacies and adverse effect profiles. In this study, GABA_A_-receptor binding affinities of CLB, *N*-desmethylclobazam (*N*-CLB, the major metabolite of CLB), CLN, and ZOL were characterized with native receptors from rat-brain homogenates and on cloned receptors from HEK293 cells transfected with combinations of α (α_1_, α_2_, α_3_, or α_5_), β_2_, and γ_2_ subtypes. Our results demonstrate that CLB and *N*-CLB have significantly greater binding affinities for α_2_- vs. α_1_-receptor complexes, a difference not observed for CLN, for which no distinction between α_2_ and α_1_ receptors was observed. Our experiments with ZOL confirmed the high preference for α_1_ receptors. These results provide potential clues to a new understanding of the pharmacologic modes of action of CLB and *N*-CLB.

## Introduction

GABA_A_ receptors are the principal inhibitory neurotransmitter-receptor system in the mammalian brain. GABA_A_ receptors are hetero-oligopentameric complexes that are members of the pentameric, ligand-gated family (also known as cys-loop family). Upon activation by the endogenous ligand GABA, receptors become permeable to chloride ions, thereby triggering chloride ion influx, which hyperpolarizes the cell and dampens activity. Most GABA_A_ receptors consist of α, β, and γ subunits, and the most prominently expressed combination is the α_1_β_2_γ_2_ receptor complex [1].

Pharmacologic modulation of GABA_A_ receptors has long been used to treat a range of conditions such as epilepsy, anxiety and panic disorders, muscle spasms, and sleep disorders. A subset of GABA_A_-receptor modulators bind to the allosteric site situated at the α- and γ-subunit interface, termed the benzodiazepine (BZD) site. The variety of possible GABA_A_-receptor subunit combinations suggests that subunit-specific compounds may allow for clinical tuning of desired therapeutic effects [2], [3], [4], [5]. Such subtype selectivity toward the GABA_A_ α_1_ subunit is linked to the clinical effectiveness of zolpidem as a sedative [6], [7]. Preclinical studies with knock-in mice carrying single amino acid point–mutations in the BZD binding site demonstrate that BZD modulation of the GABA_A_ α_1_-subunit receptors confers hypnotic/sedative effects, in addition to anticonvulsant effects. In contrast, BZD modulation of GABA_A_ α_2_–containing receptors appears to mediate anxiolytic effects and potentially may also determine anticonvulsant effect, but with no effect on sedation [2], [3], [4], [8], [9]. However, the anticonvulsant role of the GABA_A_ α_2_–containing receptors is described in the literature with divergent results.

For example, a comparative study by Fradley et al. [4] using identical drug dosages, pre-treatment times, and scorings across different mouse strains (α_1_, α_2_, and α_5_ H→R knock-in mice) found that, apparently, the α_2_ subtype plays a greater role than α_1_-containing GABA_A_ receptors in determining anticonvulsant effect following GABA_A_ receptor modulation. They address this through two models using pentylenetetrazole (PTZ)-induced seizures or tonic seizures induced by electroshock. Wild-type mice were dosage-dependently protected by diazepam in the PZT model scored using the Racine scale from 6 reversed to seizure level 1 at greatest diazepam dosage. In contrast, with the α_2_(H101R) mice, the greatest diazepam dosage produced less protection, to a scoring of approximately 3.5 on the Racine scale. No difference was observed in diazepam protection of the electroshock-induced seizures, for which a single 20-mg/kg dosage was evaluated. The authors conclude that experiments of anticonvulsant effects in rodents indicate that effect on more than one subtype is required, and that these can act synergistically [4]. Earlier work showed that the anticonvulsant action of diazepam is partially but not completely reduced in α_1_(H101R) mice [2], indicating that other GABA_A_ receptor subtypes also mediate anticonvulsant actions. Further, a study by Low et al. [10] examining diazepam-induced behavior in α_2_(H101R) and α_3_(H126R) knock-in mice revealed that the anxiolytic-like action of diazepam is absent in the α_2_(H101R), but preserved in the α_3_(H126R) mice. This suggests that the α_2_ subunit mediates this action of BZDs. In addition, the authors showed that diazepam-induced sedation and motor impairment is preserved in the two lines of mice, suggesting that other GABA_A_ subtypes mediate these effects. The anticonvulsive effects of 3-, 10-, and 30-mg/kg diazepam were assessed by percentage of mice developing tonic convulsions following PTZ administration. This yielded 100% protection at 10 and 30 mg/kg in both wild-type and α_2_(H101R) mice, while the lower dosage of 3 mg/kg resulted in convulsing in approximately 20% of the wild-type mice, but convulsing in approximately 50% of the α_2_(H101R) mice. Moreover, the α_1_ inactive compound L-838,417 that binds to α_1_ receptors but does not potentiate GABA responses retains anticonvulsant activity to PTZ and audiogenic-induced seizures in mice [3]. Collectively, the precise roles of the α_1_ and α_2_ subunits in drug-induced efficacy across preclinical models of human disease continue to be debated, except for the clear involvement of α_1_ in sedation.

Beyond the α_1_ and α_2_ subunits, the roles of BZD site modulation of the α_3_- and α_5_-containing receptor complexes are less established. Together with the α_2_ subunit, the α_3_ subunit has been implicated in BZD modulation of inflammatory and neuropathic pain responses, as well as in having some role in anticonvulsant activity [4], [11]. Negative modulation of α_5_-containing receptors has been pursued in disorders with cognitive deficits such as Down syndrome (e.g., Roche's RG-1662 currently in Phase I trials [NCT01436955 and NCT01667367]). In addition, development of tolerance to the sedative effects of diazepam (DZP) has been coupled to continued activation of α_5_ receptors [12]. The data suggest that conclusions regarding the clinical impact of significant differences in binding related to the α_3_- and α_5_-containing receptors cannot be drawn with certainty.

Clobazam (Onfi®; CLB), a structurally unique 1,5-BZD, was approved by the US Food and Drug Administration (FDA) in October 2011 for the adjunctive treatment of seizures associated with Lennox-Gastaut syndrome (LGS) in patients 2 years and older, based on results from a Phase III randomized controlled study [13]. CLB was first approved and used as an anxiolytic agent in the early 1970s [14]. Since then, the efficacy of CLB for patients with treatment-refractory epilepsy has been well-documented in a retrospective study [15]. Moreover, Lennox-Gastaut syndrome is well-known for its highly refractory nature [16]. CLB's primary active metabolite, *N*-desmethylclobazam (*N*-CLB), has a much longer half-life than the parent compound (79 h vs. 36 h), resulting in greater metabolite than CLB concentrations following prolonged dosing in humans [17]. The ratio of CLB to *N*-CLB is dependent on CYP2C19 genotypes and shows considerable correlation to polymorphisms in the CYP2C19 gene [18], [19]. Clonazepam (Klonopin®) is a 1,4-BZD used in the United States either alone or as an adjunctive treatment for LGS (petit mal variant), akinetic and myoclonic seizures [20]. Zolpidem (Ambien®), on the other hand, is approved in the United States for the short-term treatment of insomnia characterized by difficulties with sleep initiation [21].

Although drawing clinical conclusions from *in vitro* and *in vivo* animal data is not feasible, elucidating possible mechanisms of action may be an important first step in providing potential explanations of and links between preclinical and clinical findings. To examine and compare the modes of action of CLB, *N*-CLB, and CLN, we here present the results of radio-ligand binding assays to the BZD site on native receptors from the rat brain, as well as human receptors of known composition.

## Materials and Methods

### Animals

Male Lister Hooded rats from Charles River, Germany (250–300 g, 7–8 weeks) were used in these studies. All animals were housed two per cage under a 12 h light/dark cycle in a temperature- and humidity-controlled environment. Food and water were available *ad libitum*. Rats were used 1 week after arrival. The experiments were carried out at H. Lundbeck A/S, Denmark, and ethical permissions were granted by The Danish Animal Experiments Inspectorate. All animal procedures for these studies were conducted in compliance with the EC Directive 86/609/EEC and Danish law regulating experiments on animals.

### Rat-Brain Membrane Preparation

Rats were killed by rapid decapitation. Brains were rapidly removed and kept briefly on ice. The cerebellum was removed and the remaining brain tissue was homogenized in 10 volumes of buffered solution (10 mM Tris-citrate buffer [pH 7.4] and 0.32 M sucrose, 4°C) using a glass-teflon homogenizer. The homogenate was centrifuged for 10 minutes at 1,000*g* at 4°C, the supernatant collected, and the pellet re-suspended and centrifuged again. The combined supernatants were centrifuged at 13,000*g* for 20 minutes at 4°C. The pellet was re-suspended in 20–40 volumes of 5 mM Tris-citrate buffer (pH 7.4) and 5 mM EDTA solution at 4°C using an ULTRA-TURRAX® (24,000 RPM, 10–30 sec). Following 15–30 minutes of incubation on ice and 3 subsequent rounds of centrifugation (48,000*g* for 10 min at 4°C) and re-suspension (20–40 volumes of 5 mM Tris-citrate buffer [pH 7.4] and 5 mM EDTA solution at 4°C), the pellet was re-suspended in 50 mM Tris-citrate buffer (pH 7.4) and stored overnight at −20°C. On the day of the experiment, the homogenate was centrifuged (48,000*g* for 10 min at 4°C) and re-suspended (20–40 volumes of 50 mM Tris-citrate [pH 7.4] at 4°C) for a total of 4 times. For each batch of brain homogenate, the total protein content, tracer equilibrium dissociation constant (K_d_), and maximum density of receptors corrected for protein concentration (B_max_) were determined.

### Transfection and Expression of Recombinant Human GABA_A_ Receptors

Human embryonic kidney cells (i.e., HEK293) served as the host for overexpression of recombinant human GABA_A_ receptors. Specifically, α_1_, α_2_, α_3_, and α_5_ subunits were individually co-expressed with β_2_ and γ_2_ subunits in a 1∶1∶3 ratio (constructs were in the pcDNA3 vector). Batches of HEK293 cells were transiently transfected with GABA_A_-receptor cDNAs complemented with enhanced green fluorescent protein (EGFP) cDNA serving as an indicator for successful transfection. All transfections (4–6 h) were accomplished with PolyFect Transfection Reagent (QIAGEN, Denmark) in a 1∶5 weight/volume ratio (µg total cDNA to µL transfection reagent), according to the manufacturer's instructions. Cells were harvested and used for binding experiments 2–3 days after transfection.

### Cell Homogenate Preparation

The cell media was first removed followed by 3 washing steps with phosphate-buffered saline (PBS) without Ca^2+^/Mg^2+^. The cells were then harvested by scraping in PBS without Ca^2+^/Mg^2+^ and pelleted by centrifugation (3,000*g* for 5 min at 4°C). The supernatant was removed and the cell pellet was either stored at −80°C for later use or homogenized using an ULTRA-TURRAX® (24,000 rpm for 10–24 sec) in ice-cold buffer (5 mM Tris-citrate [pH 7.4] and 5 mM EDTA solution) and Protease Inhibitor Cocktail (Sigma Aldrich, Denmark). The cell-homogenate was centrifuged again (50,000*g* for 60 min at 4°C), the supernatant was discarded, and the pellet was re-suspended in buffer (50 mM Tris-citrate [pH 7.4] at 4°C). Protein content was determined using the BCA Protein Assay Reagent (Pierce/Thermo Scientific, Denmark) according to the manufacturer's instructions; and the homogenates were either stored at −80°C or used immediately for binding experiments. For each batch of cell homogenates, we determined total protein content, K_d_, and B_max_.

### Radio-Ligand Binding

All experiments utilizing cloned GABA_A_ receptors were conducted using ^3^H-flumazenil (Ro15-1788, NET 757250UC, Perkin Elmer, Denmark) and those conducted with native receptors from brain homogenates employed ^3^H-flunitrazepam (NET 567250UC, Perkin Elmer, Denmark). ^3^H-flunitrazepam was not used with cloned GABA_A_ receptors as it provided substantial non-related binding, likely from the presence of the peripheral BZD receptors in HEK293 cells (data not shown).

For each experiment, a suitable amount of cell or brain homogenate was thawed and mixed with diluted ^3^H-flumazenil or ^3^H-flunitrazepam, and the test compound of choice in a 4∶1∶1 volume ratio (typically 100 µL homogenate, 25 µL radio ligand, and 25 µL test compound). Dimethyl sulfoxide (DMSO) stock solutions were made of compounds, and these were diluted into assay buffer, with the final DMSO content kept below 0.3%. Dilutions were made using assay buffer at 4°C (for cloned receptors: 50 mM Tris-citrate [pH 7.4] and 150 mM NaCl solution; for brain homogenates: 50 mM Tris-citrate [pH 7.4]). Binding experiments were allowed to equilibrate for 90 minutes at 4°C with slow plate rotation and then harvested using a Tomtec harvester (Tomtec, Inc., CT, USA) with harvest buffer (50 mM Tris-citrate [pH 7.4] at 4°C) onto 96-well format glass-fiber filters (B-size filters) pre-wetted with 0.1% polyethyleneimine. The degree of retained radioactivity was quantified on a Wallac MicroBeta counter (Wallac/Perkin Elmer, Denmark). On each 96-well plate, controls for total binding (assay buffer) and non-specific binding (100 µM and 20 µM DZP for cloned receptors and brain homogenates, respectively) were included, allowing for calculations of the relative percentage inhibition. The time of radioactivity counting was set so the total counts per well were >10,000.

Saturation experiments to determine K_d_ and B_max_ were performed with 12 different concentrations of tracer (up to 10 nM). Specific binding at the K_d_ concentration was >80% and depletion of tracer <10% in all cases. Half-maximum inhibitory concentrations (IC_50_) for test compounds were determined through 10-point, serial 5-fold dilutions covering at least 7 log scales surrounding the experimentally determined IC_50_ values. Individual determinations for each compound on each GABA_A_-receptor subtype or brain homogenate were made on independent experimental days and were conducted with a tracer concentration close to the K_d_ value.

### Data Analysis

K_d_ and B_max_ values were determined through saturation binding experiments and fitting the specific binding signal (total binding minus the background binding obtained with DZP) to a hyperbolic function (Equation 1).
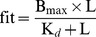
(1)where L is the concentration of the radio-ligand. K_d_ and B_max_ were presented as the average values from several experiments. The experimentally determined concentration-inhibition data were fitted to the 4 Parameter Logistic (4PL) model (Equation 2) using non-linear regression (Microsoft Excel and XLfit) to yield the IC_50_ values.
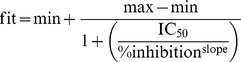
(2)The IC_50_ values were transformed to K_i_ (binding affinity) values using the Cheng-Prusoff correction (Equation 3).
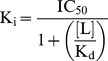
(3)where L is the concentration of the radio-ligand. The individual K_i_ values were transformed into pK_i_ (pK_i_ = −log[K_i_]) and presented as mean values with standard errors of the mean (SEM). The mean of the pK_i_ values were evaluated for statistical differences across the receptor subtypes per compound by one-way ANOVA with Tukey's Multiple-Comparison *post-hoc* Test (*P*<0.05 considered significant). Testing the hypothesis of same mean across subtypes was done using GraphPad Prism 4 (GraphPad Software, Inc., La Jolla, CA).

## Results

**Figure 1:**
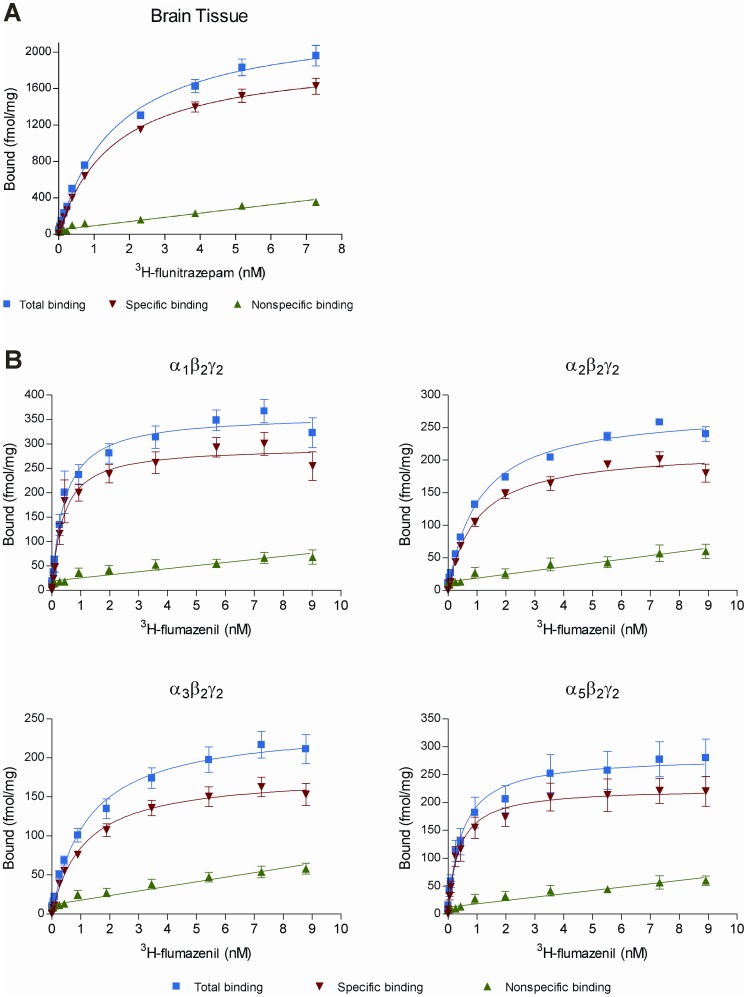
Saturation binding experiments of equilibrium binding of various concentrations of radio-ligand. (A) Rat brain homogenate with ^3^H-flunitrazepam, and (B) recombinant human GABA_A_ receptors with ^3^H-flumazenil.Total binding, non-specific binding, and specific binding are shown.

K_d_ and B_max_ values for ^3^H-flunitrazepam (used as a tracer for binding) on rat-brain homogenates were 1.5±0.2 nM and 1,906±166 fmol/mg, respectively (Figure 1A). Specific binding at the K_d_ value was determined to be 85%±3.3% (N = 6). CLN was determined to have subnanomolar binding affinity (0.26 nM), whereas CLB and *N*-CLB exhibited submicromolar binding affinities (151 and 133 nM, respectively, Table 1).

**Table 1:** Clonazepam, clobazam, and *N*-desmethylclobazam binding affinities for rat-brain homogenates using ^3^H-flunitrazepam as tracer.

	pK_i_ (mean ±SEM)	K_i_ (nM)	N
**Clonazepam**	9.58 (±0.08)	0.26	4
**Clobazam**	6.82 (±0.10)	151	4
***N*** **-desmethylclobazam**	6.88 (±0.17)	133	4

K_i_ = binding affinity; SEM = standard error of the mean.

Transfected HEK293 cell homogenates were probed with ^3^H-flumazenil as the tracer; K_d_ values were 0.43–1.24 nM and B_max_ values were 188–300 fmol/mg across the 4 GABA_A_-receptor complexes (Figure 1B and Table 2). These K_d_ values are in agreement with previously published data for combinations of α_1_, α_2_, α_3_, and α_5_ subunits with β_3_ and γ_2_ expressed in mouse fibroblast L(tk^−^) cells [22].

**Table 2:** Saturation equilibrium binding results of ^3^H-flumazenil on four different GABA_A_-receptor complexes overexpressed in HEK293 cells.

	α_1_β_2_γ_2_	α_2_β_2_γ_2_	α_3_β_2_γ_2_	α_5_β_2_γ_2_
^3^H-flumazenil	Mean (±SEM)	Mean (±SEM)	Mean (±SEM)	Mean (±SEM)
**B_max_ (fmol/mg)**	300 (±22)	225 (±11)	188 (±18)	227 (±24)
**K_d_ (nM)**	0.50 (±0.05)	1.11 (±0.12)	1.24 (±0.14)	0.43 (±0.05)

B_max_ = maximum density of receptor binding sites; K_d_: tracer equilibrium dissociation constant; SEM = standard error of the mean.

The number of experiments ranged from 9 to 12.

**Figure 2:**
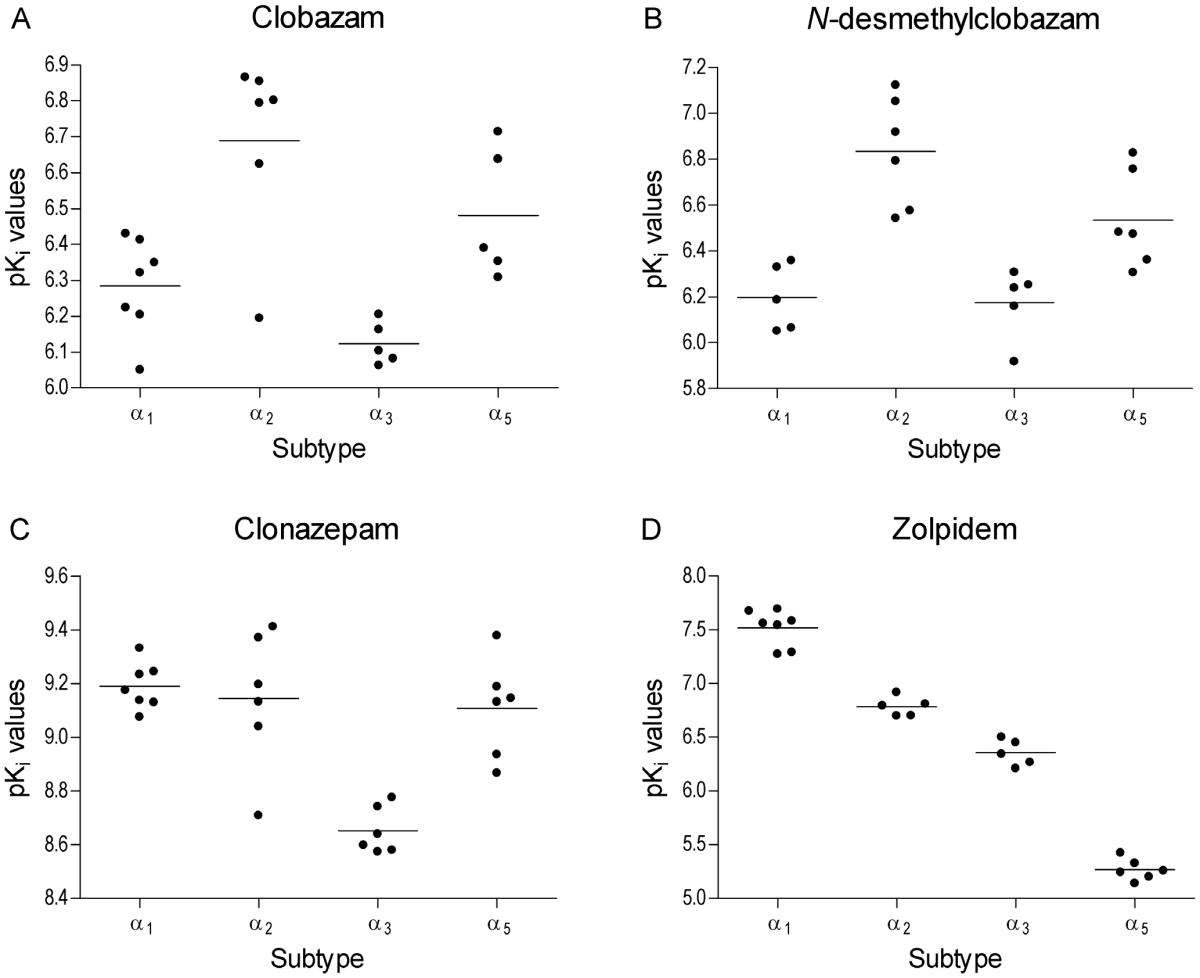
Distribution of individually determined pK_i_ values for (A) clobazam, (B) *N*-desmethylclobazam, (C) clonazepam, and (D) zolpidem across GABA_A_-receptor subtypes.

All 4 compounds fully displaced ^3^H-flumazenil in a concentration-dependent manner, consistent with competitive binding to the BZD binding site on GABA_A_ receptors. CLB displaced ^3^H-flumazenil at submicromolar K_i_ values across all α-subunit subtypes tested in the range of 205 nM to 753 nM (α_2_<α_5_<α_1_<α_3_). In addition, submicromolar K_i_ values in the interval of 147 nM to 668 nM were determined for *N*-CLB (α_2_<α_5_<α_1_<α_3_, Table 3). CLN and ZOL were found to have K_i_ values ranging from 0.65–2.20 nM (α_1_<α_2_<α_3_<α_5_) and 30–5,431 nM (α_1_<α_2_<α_5_<α_3_), respectively (Table 3), with ZOL showing preferential binding at α_1_. These results for ZOL and CLN are in agreement with published data [23], [24]. The range of pK_i_ values for all 4 compounds are depicted in Figure 2, and the results (i.e., *P*-values) of pair-wise comparisons of the mean pK_i_ values between receptor subtypes performed with Tukey's Multiple-Comparison Test are provided in Table 4. The binding profiles across the receptor subtypes are different for these compounds (i.e., both CLB and N-CLB show significantly greater binding affinities for α_2_ over α_1_, whereas CLN does not show this particular subtype difference). In line with its use as a sedative, ZOL has the greatest affinity for α_1_ versus all other receptor subtypes.

**Table 3:** Binding affinities of clobazam, *N*-desmethylclobazam, clonazepam, and zolpidem to four different human GABA_A_-receptors complexes expressed in HEK293 cells obtained by displacement of ^3^H-flumazenil.

	α_1_β_2_γ_2_			α_2_β_2_γ_2_			α_3_β_2_γ_2_			α_5_β_2_γ_2_		
	pK_i_			pK_i_			pK_i_			pK_i_		
Drug	Mean (±SEM)	N	K_i_ (nM)	Mean (±SEM)	N	K_i_ (nM)	Mean (±SEM)	N	K_i_ (nM)	Mean (±SEM)	N	K_i_ (nM)
Clobazam	6.28 (±0.05)	7	519	6.69 (±0.11)	6	205	6.12 (±0.03)	5	753	6.48 (±0.08)	5	331
*N*-desmethylclobazam	6.20 (±0.06)	5	634	6.83 (±0.10)	6	147	6.18 (±0.07)	5	668	6.53 (±0.09)	6	292
Clonazepam	9.19 (±0.03)	7	0.65	9.14 (±0.10)	6	0.72	8.65 (±0.04)	6	2.2	9.11 (±0.08)	6	0.78
Zolpidem	7.52 (±0.05)	7	30	6.78 (±0.04)	5	165	6.35 (±0.05)	5	442	5.27 (±0.04)	6	5431

K_i_ = binding affinity; SEM = standard error of the mean.

**Table 4:** Binding affinities normalized to GABA_A_ α_2_ (ratios of K_i_ values, show in gray) and pair-wise comparison of the mean pK_i_ values of clobazam, *N*-desmethylclobazam, clonazepam, and zolpidem across GABA_A_ receptor subtypes.

Clobazam	α_1_β_2_γ_2_	α_2_β_2_γ_2_	α_3_β_2_γ_2_	α_5_β_2_γ_2_
**α_1_β_2_γ_2_**	2.5	*P*<0.01	NS	NS
**α_2_β_2_γ_2_**	–	1	*P*<0.001	NS
**α_3_β_2_γ_2_**	–	–	3.7	*P*<0.05
**α_5_β_2_γ_2_**	–	–	–	1.6

*N*-desmethylclobazam	α_1_β_2_γ_2_	α_2_β_2_γ_2_	α_3_β_2_γ_2_	α_5_β_2_γ_2_
**α_1_β_2_γ_2_**	4.3	*P*<0.001	NS	*P*<0.05
**α_2_β_2_γ_2_**	–	1	*P*<0.001	NS
**α_3_β_2_γ_2_**	–	–	4.6	*P*<0.05
**α_5_β_2_γ_2_**	–	–	–	2.0

Clonazepam	α_1_β_2_γ_2_	α_2_β_2_γ_2_	α_3_β_2_γ_2_	α_5_β_2_γ_2_
**α_1_β_2_γ_2_**	0.9	NS	*P*<0.001	*NS*
**α_2_β_2_γ_2_**	–	1	*P*<0.001	NS
**α_3_β_2_γ_2_**	–	–	2.2	*P*<0.001
**α_5_β_2_γ_2_**	–	–	–	1.1

Zolpidem	α_1_β_2_γ_2_	α_2_β_2_γ_2_	α_3_β_2_γ_2_	α_5_β_2_γ_2_
**α_1_β_2_γ_2_**	0.2	*P*<0.001	*P*<0.001	*P*<0.001
**α_2_β_2_γ_2_**	–	1	*P*<0.001	*P*<0.001
**α_3_β_2_γ_2_**	–	–	2.7	*P*<0.001
**α_5_β_2_γ_2_**	–	–	–	33

NS = not significant.

The degree of significance from the comparison of the mean of the pK_i_ values following one-way ANOVA with Tukey's multiple-comparison *post-hoc* test is presented across the subtypes per compound.

## Discussion

This report describes the binding affinities of a set of compounds (clobazam [CLB], *N*-desmethylclobazam [*N*-CLB], clonazepam [CLN], and zolpidem [ZOL]) to native GABA_A_-receptor complexes overall (obtained from rat-brain homogenates) and to specific human GABA_A_-receptor subunits (obtained from HEK293 cells transiently transfected with human cDNA encoding GABA_A_-receptors, namely α_1_β_2_γ_2_, α_2_β_2_γ_2_, α_3_β_2_γ_2_, or α_5_β_2_γ_2_). CLB and *N*-CLB were found to have similar submicromolar binding affinities for native receptors, and CLN was found to display subnanomolar binding affinity. Data from our head-to-head comparisons on human receptors demonstrate that the 1,5-BZD CLB, and its active metabolite *N*-CLB, have significantly greater affinities for the α_2_-receptor subtype versus the α_1_-receptor subtype, whereas the 1,4-BZD CLN does not. This subtype preference was slightly more marked for *N*-CLB than for CLB. With the same experimental conditions, the sedative agent ZOL displayed the greatest affinity for α_1_-containing receptor complexes. The results from these experiments confirm that our assay is sensitive in determining differences in subtype affinity. Importantly, the binding affinities observed for the 2 different sources of GABA_A_ receptors (rat-brain homogenates and transfected HEK293 cells) were in the same concentration range for the tested compounds. Therefore, the recombinant receptors are representative of the native receptors. The K_i_ values for CLB on native receptors are in the same range as previously published data [25], whereas our determined affinity for *N*-CLB is markedly different than what this previous group has published. We can only conjecture as to what may have caused this difference in binding affinity for *N*-CLB. Arendt et al. [25] unfortunately do not present information on the variation of their results and nor on the K_d_ or B_max_ values obtained for ^3^H-flunitrazepam. Hence, evaluation of the variation and significance of their results is not possible. We note that the affinities we found using native receptors are in the same range as those we found using recombinant receptors. Interestingly, Haigh et al. [26] reported that *N*-CLB is effective both in a preclinical convulsion model in mice, but also in human patients when administered at dosages leading to concentrations similar to those obtained following dosing of clobazam (i.e., 8 of 9 patients responded favorably to *N*-CLB).

Upon initiation of CLB or CLN in clinical settings, drug concentrations in the human brain and plasma rise gradually. Extrapolating our preclinical binding data to such a clinical setting would imply that CLB (and resulting *N*-CLB) will initially bind to the α_2_ receptor before interacting with α_1_ receptor because of the difference in binding affinities. CLN, on the other hand, will bind simultaneously to both the α_1_ receptor and the α_2_ receptor by virtue of identical binding affinities for the two receptors. With the assumption that similar percentages of binding to α_2_ receptors are obtained during antiepileptic treatment with CLB and CLN, our data suggest that a separation to α_1_-receptor binding may be present for CLB but not for CLN.

During prolonged clinical use of CLB, concentrations of *N*-CLB build up (a direct result of its longer half-life) to more than 2-fold of the parent compound [27]. In this situation, given their similar K_i_ values, both compounds potentially would interact with GABA_A_ receptors and act together to produce a combined modulatory effect. In addition, the significant separation between α_1_ and α_2_ for CLB/*N*-CLB could increase during *N*-CLB accumulation following prolonged dosing, as *N*-CLB presents the largest K_i_-value ratio of α_1_ to α_2_ (Table 4).

Our results are interesting in the light of data from published studies comparing the clinical effects of orally administered CLB and CLN [28], [29], [30] for use for acute conditions. These independent double-blind, placebo-controlled (in the case of [30], an active placebo was used), cross-over studies of healthy volunteers show a greater incidence of sedation following oral dosing with CLN (0.5, 1 or 2 mg) relative to CLB (10 or 20 mg) [28], [29], [30]. The FDA-recommended starting oral dosages of CLB for patients with LGS weighing more than 30 kg are 10 mg/day [31]. The FDA-recommended initial oral dosage of CLN for adult patients with seizure disorders is 1.5 mg/day [20]. While the clinical data are very limited, preclinical data from a mice study suggest greater specificity of CLB and *N*-CLB for anticonvulsive/antiepileptic over sedative effects relative to 1,4-BZDs [32]. Findings from the spontaneously epileptic Ihara rat also suggest differential effects of CLB relative to CLN, with respect to differences in antiepileptic and sedative effects [33].

In summary, our binding studies with GABA_A_-receptor complexes expressing different α-subunit subtypes show that CLB and *N*-CLB have significantly greater affinities for the α_2_-containing receptors over α_1_-containing receptors. On the other hand, CLN has similar affinities for both α_1_- and α_2_-containing receptors, and, as previously shown, ZOL has greatest affinity for α_1_-containing receptors. This data set presents information on one important aspect of BZD interaction with GABA_A-_receptors (i.e., affinity), but our work did not investigate another important aspect of BZD function (i.e., efficacy — the degree of potentiation of the GABA response across the subtypes) [5]. To gain a full understanding of the modulatory effect of these compounds and the differences across subtypes we report here, such experiments should be conducted.
